# Supplementary material for: Comparison of Different Label-Free Techniques for the Semi-Absolute Quantification of Protein Abundance
Source: Proteomes. 2022 Jan 7;10(1):2. doi: 10.3390/proteomes10010002 (PMC8788469; doi:10.3390/proteomes10010002)
Supplement: Supplementary file 1 [file proteomes-10-00002-s001.zip › Supplementary_figures_methods.pdf]

Comparison of different label-free techniques for the semi-absolute quantification of protein abundance

Aaron Millan-Oropeza[1], Mélisande Blein-Nicolas[2], Véronique Monnet[1], Michel Zivy[2], Céline Henry[1]

[1]PAPPSO, Université Paris-Saclay, INRAE, AgroParisTech, Micalis Institute, 78350, Jouy-en-Josas, France

[2]PAPPSO, Université Paris-Saclay, INRAE, CNRS, AgroParisTech, GQE - Le Moulon, 91190, Gif-sur-Yvette, France

KEYWORDS: label free, metabolic models, *Saccharomyces*, semi-absolute quantification, quantitative proteomics, TPA, UPS2

SUPPLEMENTARY METHODS

Total proteins extraction and *in gel* digestion

Cells pellets obtained from 30 ml of yeast cultures were washed twice with 50 mM TRIS-HCl pH=7.8 and suspended in 3 ml of lysis buffer containing : 6 M urea (Sigma, U5378), 2 M thiourea (Sigma, T8656), 5 mM dithiothreitol (DTT, Sigma) and 0.1 M TRIS-HCl pH=8. Aliquots were supplemented with 150 µl of prepared proteases inhibitor cocktail (Sigma, P8465). Cells lysis was conducted using a cell disruptor (Constant systems Ltd. One shot model) at 2.4 Kbars and cells debris were removed by centrifugation (15 min at 4000 g, 4° C). Soluble protein concentrations were measured according to the 2-D Quant kit protocol (GE Healthcare Life Sciences, 80-6483-56). Aliquots of 15 µg of total protein extract were separated using one-dimensional SDS-PAGE short migration gels (1x1 cm lanes, Invitrogen, NP321BOX). Digestion was performed on excised bands from stacked gels. Reduction was carried in DTT solution at 10 mM for 30 minutes at 56 °C. The extracts were alkylated with iodoacetamide (IAA) solution at 55 mM for 45 min in darkness at room temperature (RT). Samples were first digested for 3 h at 37 °C by adding 300 ng of Lysyl-Endopeptidase (Wako, 125-05061). Then, a second digestion was performed with 300 ng of sequencing-grade modified trypsin (Promega) overnight at 37 °C. To quench digestion, the pH was adjusted to 2 by adding 2 µl of TFA 30% (v/v). Supernatants were recovered and the peptides were extracted with a mixture of 0.5% trifluoroacetic acid (TFA) and 50% acetonitrile (ACN) in water. Extracted tryptic peptides were dried in vacuum and resuspended in 75 µl of loading buffer containing 0.08% (v/v) of trifluoroacetic acid (TFA) and 2% (v/v) of acetontrile (ACN) in water for mass spectrometry analysis.

UPS2 standard sample preparation

The UPS2 standard (Sigma) contains 48 human proteins with different molecular weights (from 6 to 83 KDa) distributed on 6 molar concentrations ranging from 50 pmoles to 500 amoles. The content of one vial of UPS2 standard (10.6 µg) was resuspended in 35 µl of lysis buffer (see total proteins extraction protocol) and incubated for 2 h at RT to perform the reduction step. Alkylation was carried out with freshly prepared IAA solution (50 mM final concentration) for 45 min in darkness. Digestion was conducted first with 353 ng of Lysyl-Endopeptidase (Wako) incubated during 3 h at 37 °C. Samples were diluted 1:6 with milliQ water and supplemented with 353 ng of Trypsin (Promega), incubation occurred overnight at 37 °C. To quench digestion, the pH was adjusted to 2 by adding 2 µl of TFA 30% (v/v). The peptide mixtures were cleaned with ZipTips C18 systems (Millipore) by

applying three washing cycles (solution containing 3% (v/v) ACN and 0.06% (v/v) glacial acetic acid). Elution of peptides was achieved using a buffer with 40% (v/v) ACN and 0.06% (v/v) glacial acetic acid. The extracted tryptic peptides were dried on vaccum and resuspended in 25 µl of loading buffer.

Mass spectrometry analysis

MS analyses were performed on a Dionex U3000 RSLC system coupled to an Orbitrap Fusion™ Lumos™ Tribrid™ mass spectrometer (Thermo Fisher Scientific). For each sample, 4 µl of digested peptides were injected and separated using a packed column Acclaim™ PepMap™, 75 µm x 500 mm, C18, 3 µm, 100 Å (Thermo Fisher Scientific). Buffer A consisted of 0.1% formic acid in 2% ACN and buffer B of 0.1 % formic acid in 80% ACN. Peptide separation was achieved at 300 nl/min with a linear gradient from 4 to 35% buffer B for 160 min and 35% to 50% for 10 min. One run took 180 minutes including the regeneration step at 98 % buffer B. Ionization (1.6 kV ionization potential) and capillary transfer (275 °C) were performed with a liquid junction and a capillary probe (SilicaTip™ Emitter, 10 µm, New Objective). MS/MS analysis was performed in data dependent acquisition mode. The mass spectrometer acquisition settings were set as follows. Full MS scan in Orbitrap (scan range [m/z] = 400–1600) with a resolution of 120,000 (AGC target = 5×10^5, max. injection time of 100 ms, data type = centroid). Analyzed charge states were set in Top speed mode with a cycle of 3 s for the most intense double or triple charged precursor ions. The dynamic exclusion was during 60 s. Ions in each MS scan over threshold 50,000 were selected for fragmentation (MS2). MS2 was performed using High Collision Dissociation (HCD) mode in the Orbitrap with resolution of 15,000 (30 % collision energy, AGC target of 5.0×10^4 and max. injection time of 54 ms). Polysilaxolane ions m/z 445.12002, 519.13882, 593.15761 and 667.1764 were used for internal calibration.

Custom database construction

A custom database was constructed by matching the orthologs proteins between the genomes of the experimental strain *S. cerevisiae* CEN.PK113-7D (UNIPROT, accessed on 14/09/2017) and the reference strain *S. cerevisiae* s288c (accessed on UNIPROT, 03/11/2017), this was motivated by the need of protein IDs from the reference for further data parsing. Unique labels (CHASSY_ID) were attributed to proteins encoded by orthologous genes that fulfilled a minimum percentage of identity (90 %) and a minimum HSP coverage (80 %) after BLAST RBH, specific proteins were also kept for further analysis.

Intra-sample accuracy assessment

The accuracy intra-sample was evaluated in the seven quantification methods (PAI, emPAI, SAF, NSAF, IBAQ, TOP3 and SUMnorm) and in both strategies to convert relative abundance into absolute values (using a UPS2 calibration line and with TPA). To the bulk samples spiked with UPS2 proteins, one UPS2 protein was removed from the dataset and its semi-absolute quantification was determined either with the calibration line resulting from the remaining UPS2 proteins or with the TPA. This action was repeated for each UPS2 protein on each method in order to determine the absolute error for the measurements of the UPS2 proteins.

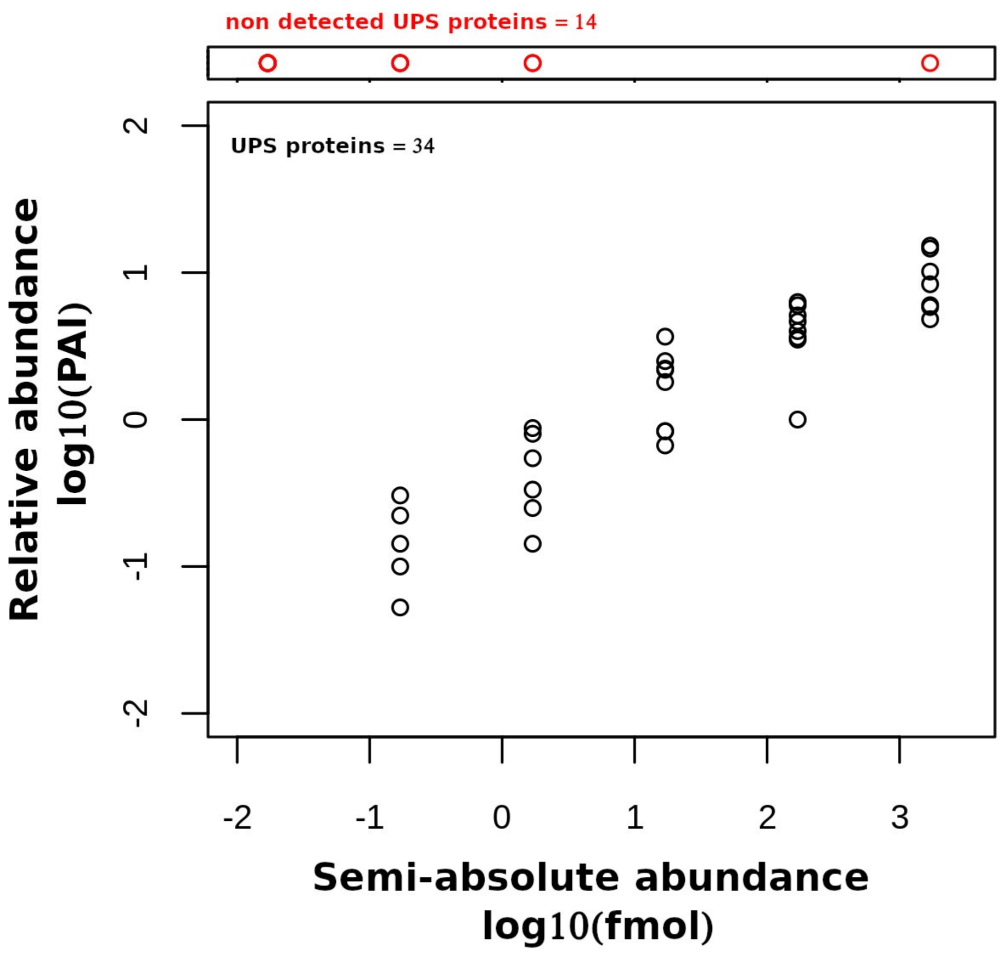

Figure S1. Detected UPS2 proteins in 400 ng (pure UPS2 standard) analyzed in a single MS run (34/48) in an Orbitrap™ Fusion™ Lumos Tribrid™. Non-identified proteins (in red) corresponded to different molar concentrations of the UPS2 standard: 0.017 fmol (P05413, P10636, P02788, P08758, P02741, P10145, P00441, P01375), 0.17 fmol (P01579, P51965, P99999), 1.7 fmol (P01344, P01127) and 1700 fmol (P02768).

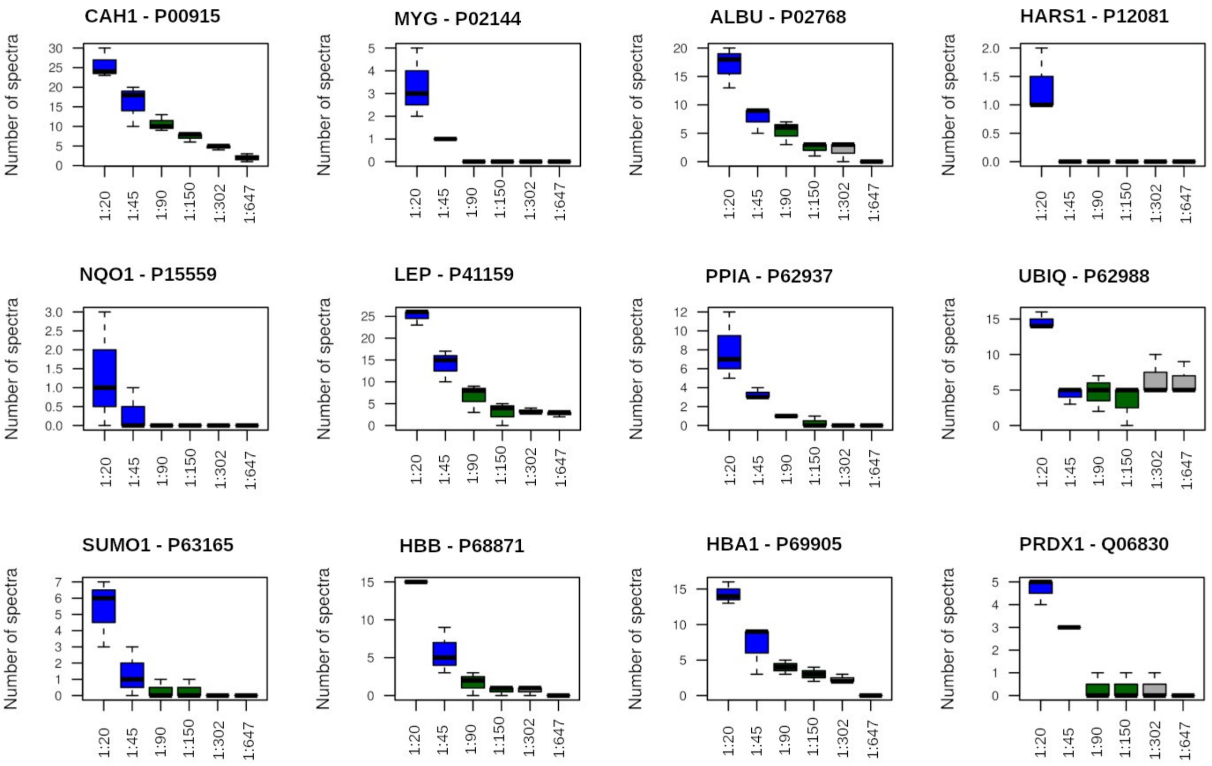

Figure S2. Spectral counts of UPS2 proteins detected when spiked into the bulk samples at different ratios of UPS2:yeast. The ratios (ups2:YEAST) were 1:20, 1:45, 1:90, 1:150, 1:302 and 1:647.

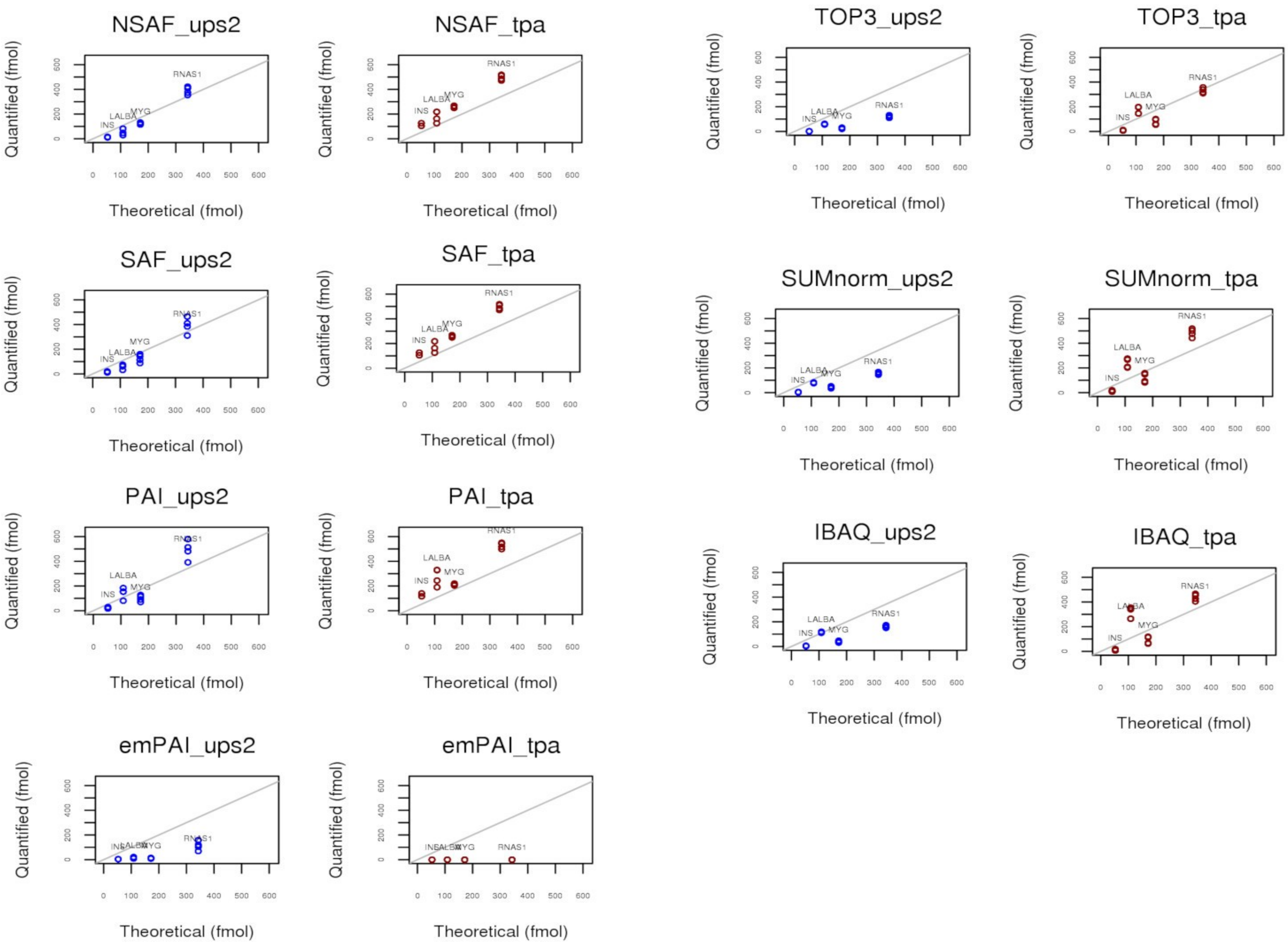
NSAF_ups2
NSAF_tpa
TOP3_ups2
TOP3_tpa
SAF_ups2
SAF_tpa
SUMnorm_ups2
SUMnorm_tpa
PAI_ups2
PAI_tpa
IBAQ_ups2
IBAQ_tpa
emPAI_ups2
emPAI_tpa
Quantified (fmol)
Theoretical (fmol)
INS
LALBA
MYG
RNAS1

Figure S3. Comparison of different methods to assess semi-absolute quantification in the purified proteins at known concentrations. The purified proteins are: Insulin from bovine pancreas (INS) at 51.9 fmol, Alpha-lactalbumin from bovine milk (LALBA) at 108.6 fmol, Myoglobin from equine skeletal muscle (MYG) at 181.8 fmol and Ribonuclease A from bovine pancreas (RNAS1) at 342.6 fmol (n=4). The different methods of quantification are: i) Normalized Spectral Abundance Factor (NSAF), ii) Spectral Abundance Factor (SAF), iii) Protein Abundance Index (PAI), iv) Exponentially Modified PAI (emPAI), v) Intensity-Based Absolute Quantification (IBAQ), vi) top three most intense peptides (TOP3), and vii) Sum of XICs normalized by protein length (SUMnorm). The two strategies to convert relative quantification into absolute abundance are: i) using the spiked UPS2 proteins to calculate a calibration curve (UPS2), and ii) using the Total Protein Approach (TPA).

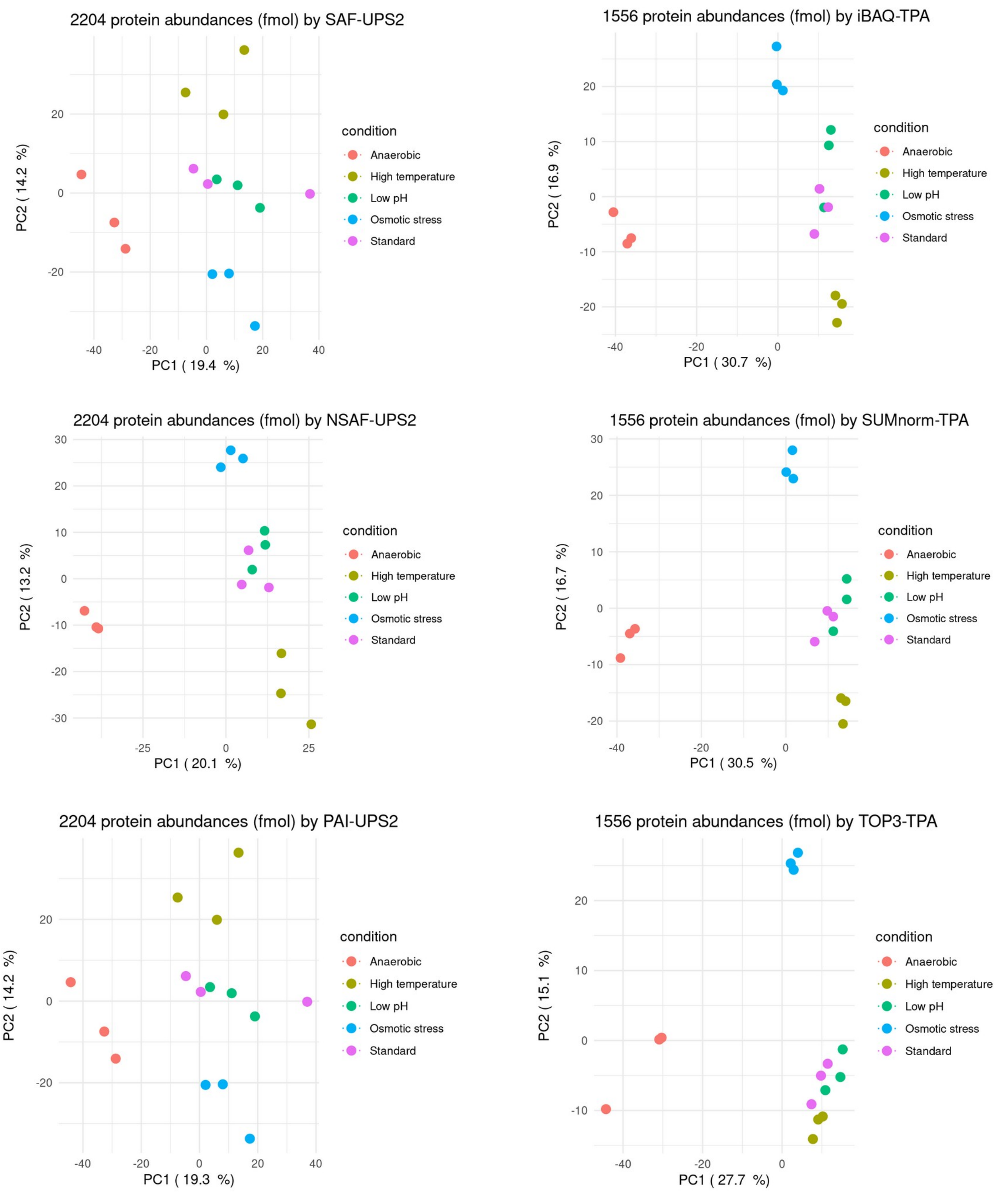

Figure S4. Principal Component Analysis (PCA) on yeast proteins expressed in absolute values (fmol). Data were obtained from the selected SC-based methods (UP2 approach) quantification methods NSAF, SAF and PAI. As well as the selected XIC-based methods (TPA) iBAQ, TOP3 and SUMnorm.
