# Supplementary figures and images for: Comparison of Different Label-Free Techniques for the Semi-Absolute Quantification of Protein Abundance
Source: Proteomes. 2022 Jan 7;10(1):2. doi: 10.3390/proteomes10010002 (PMC8788469; doi:10.3390/proteomes10010002)

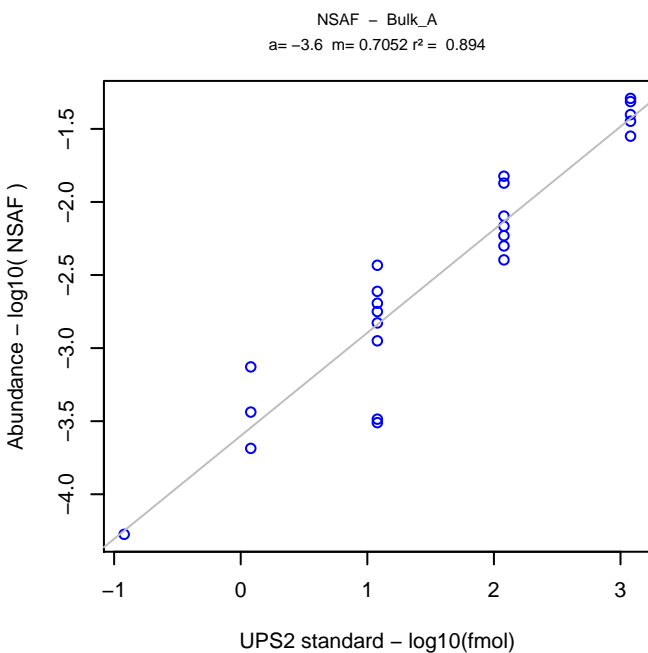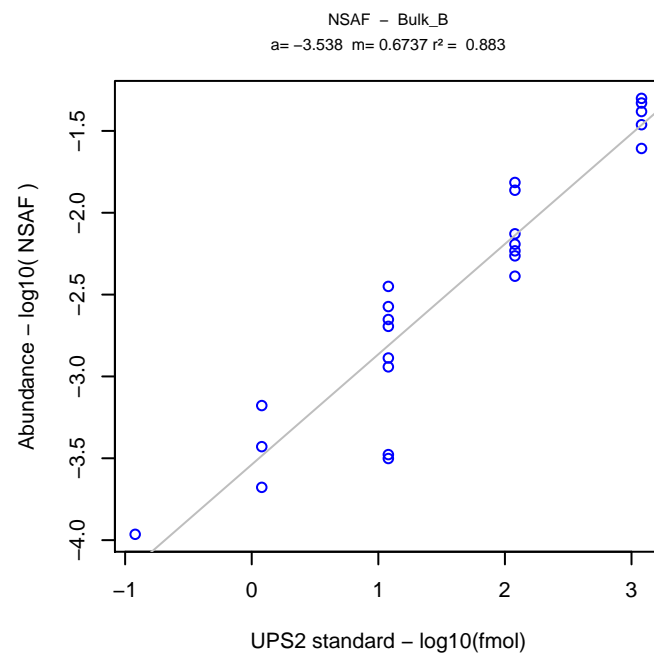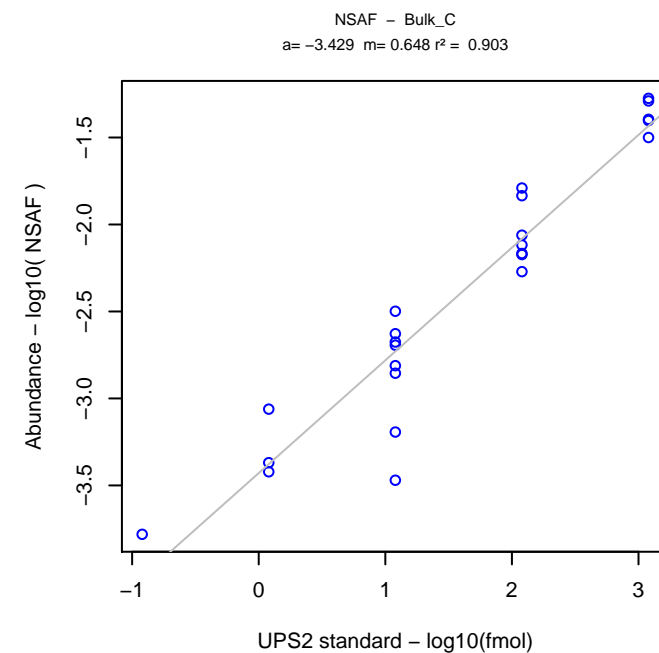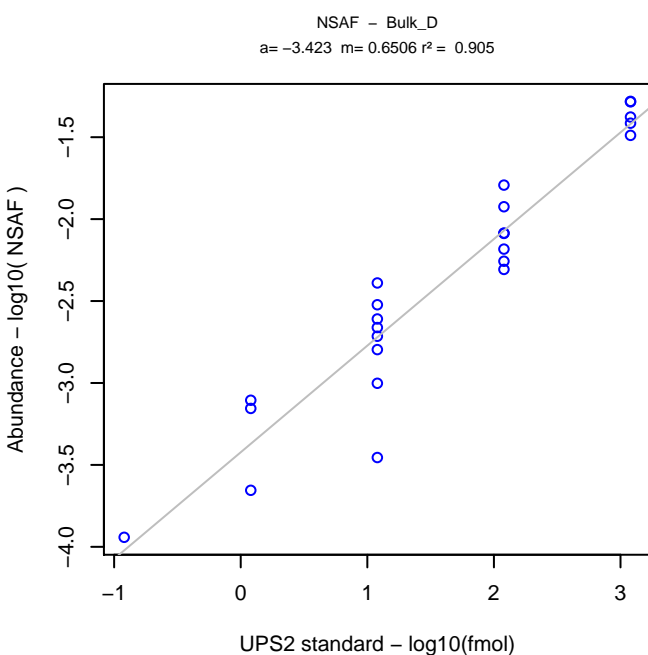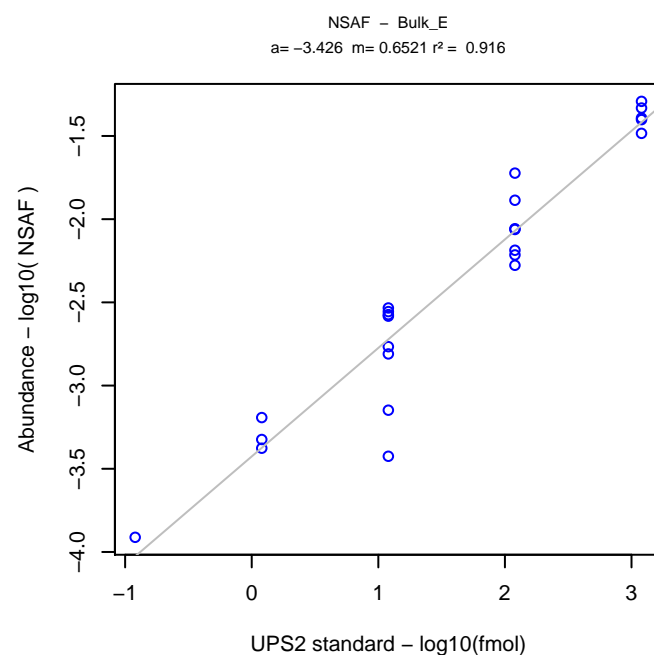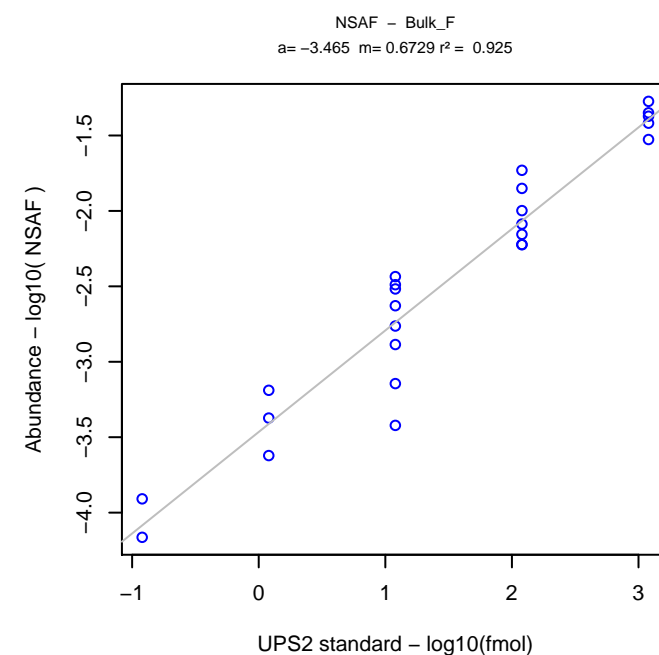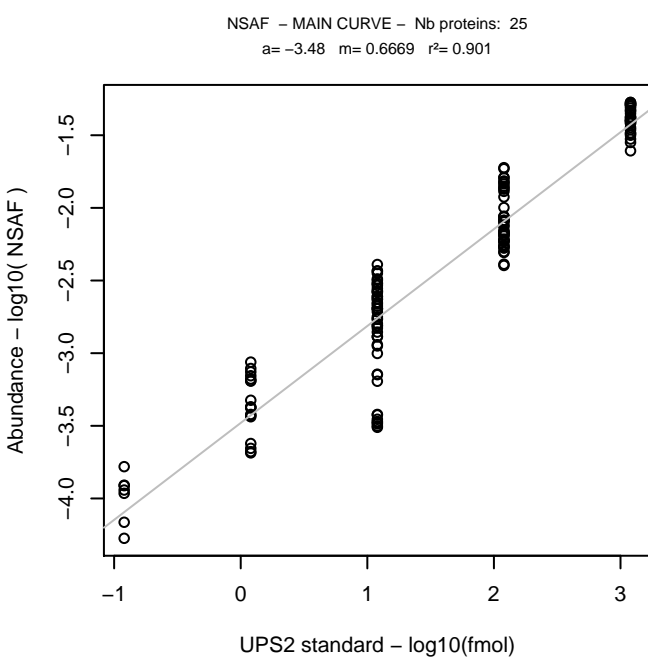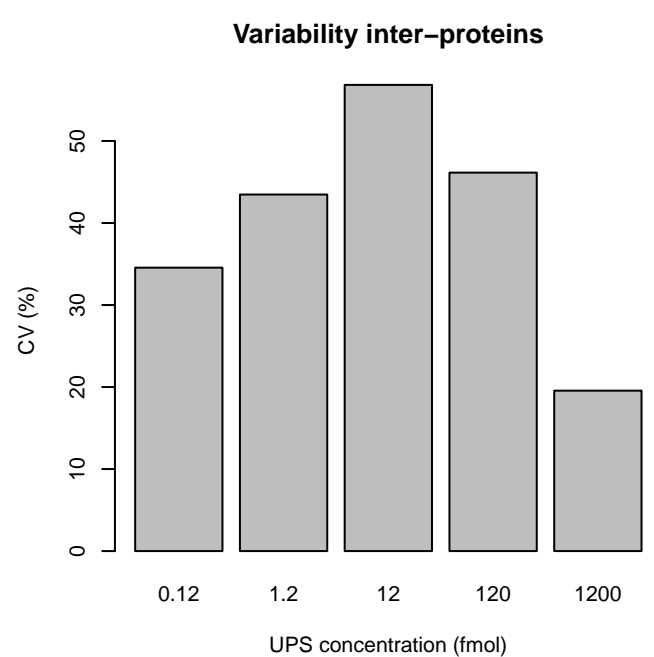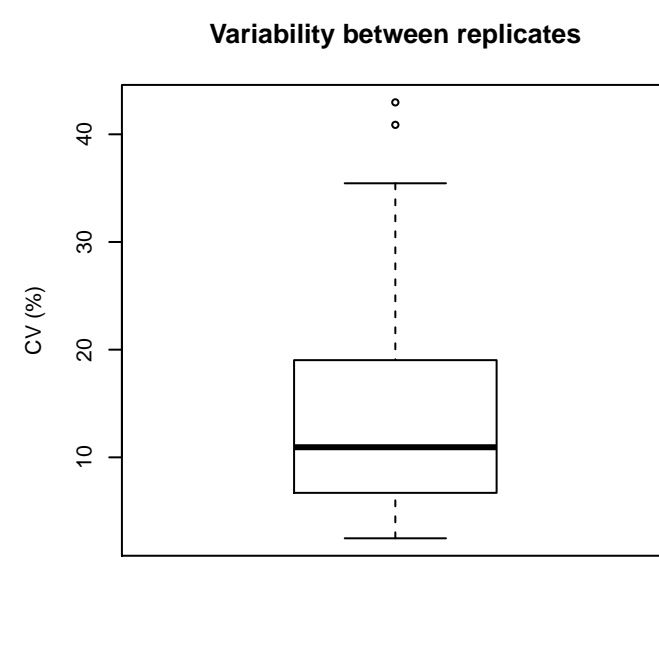

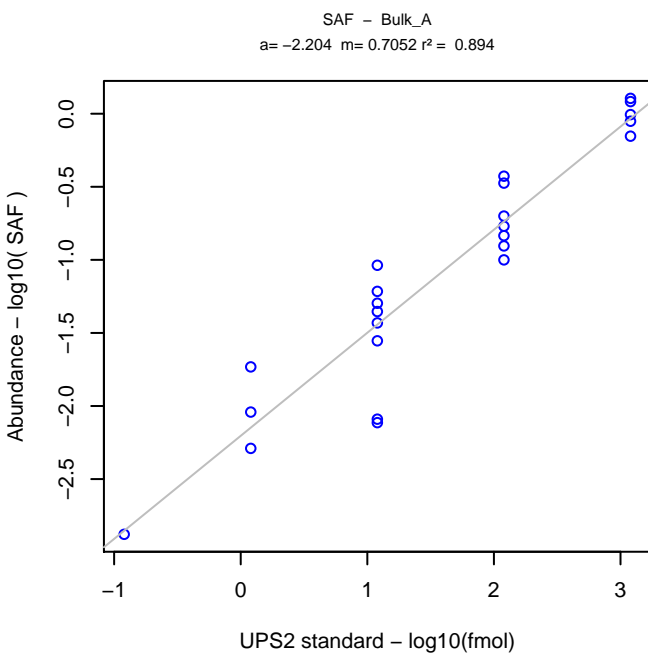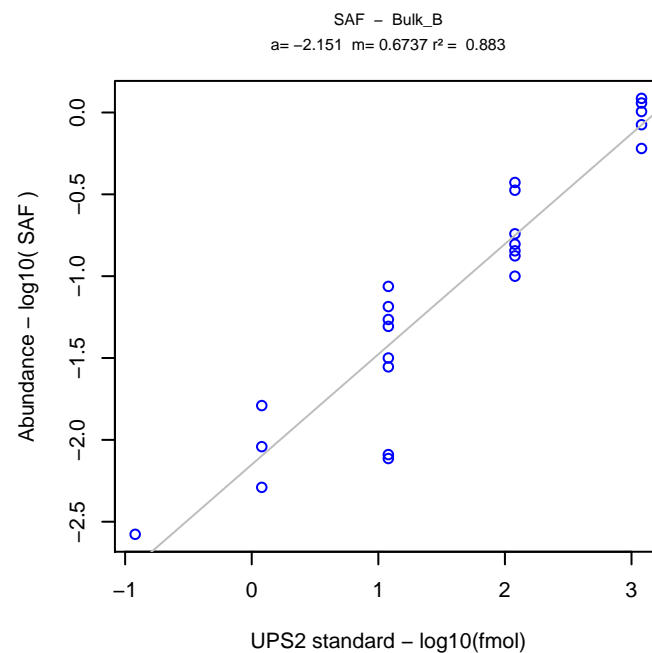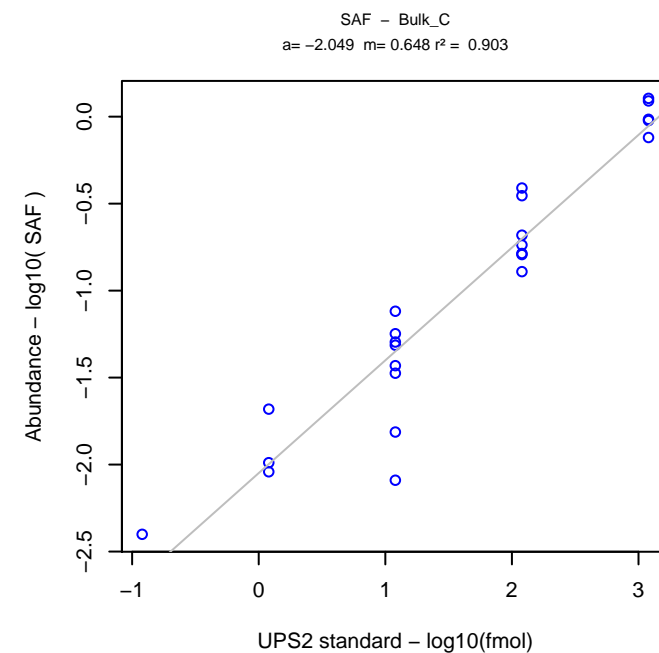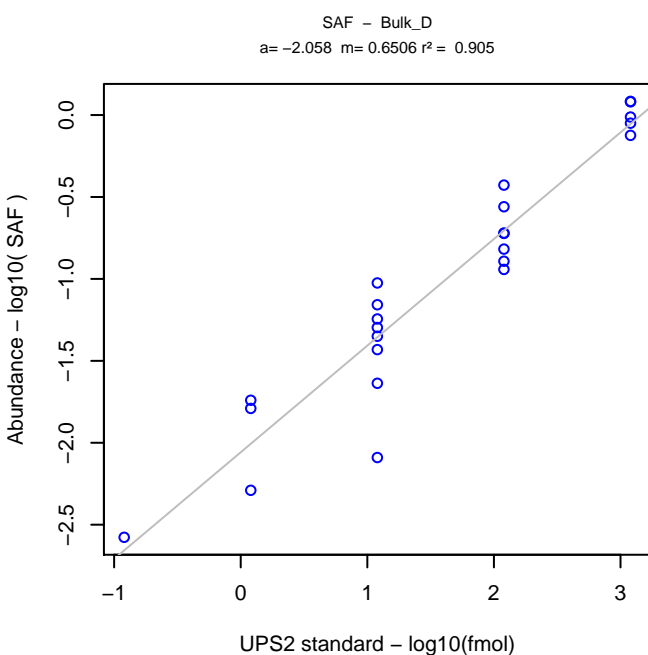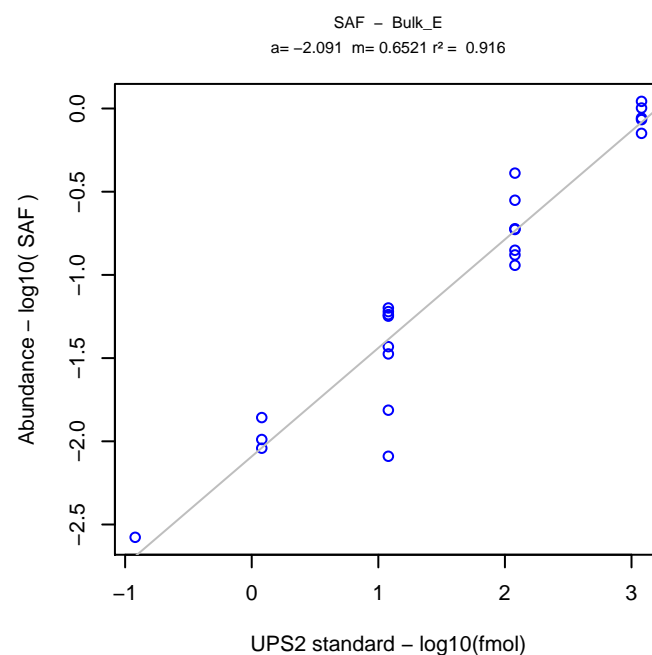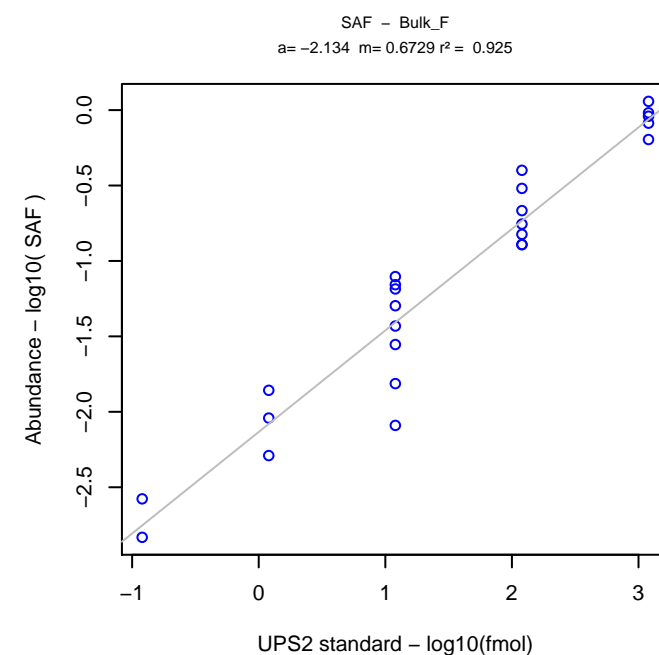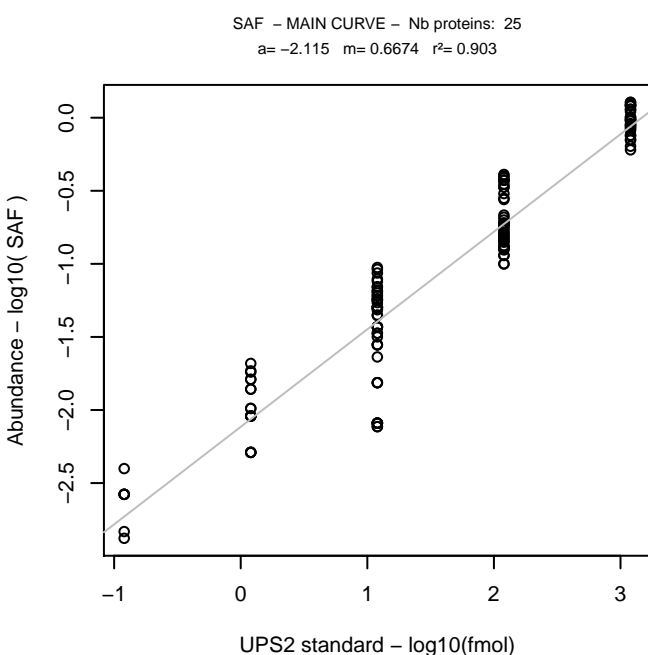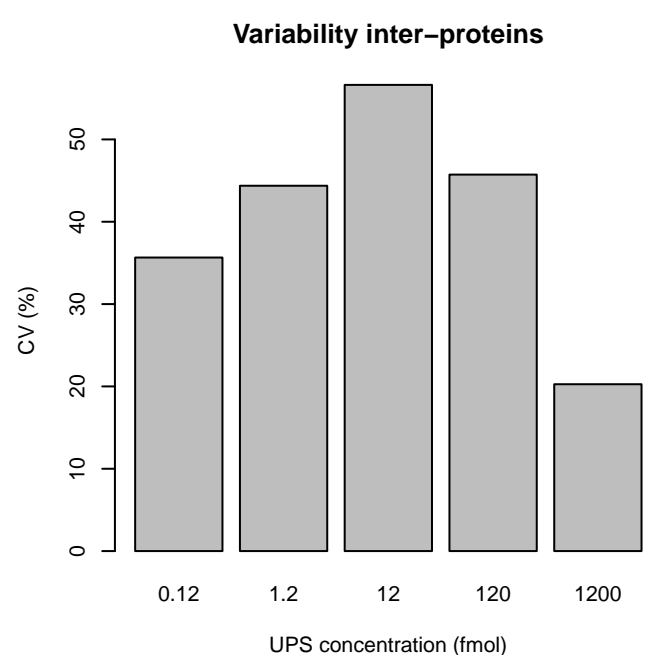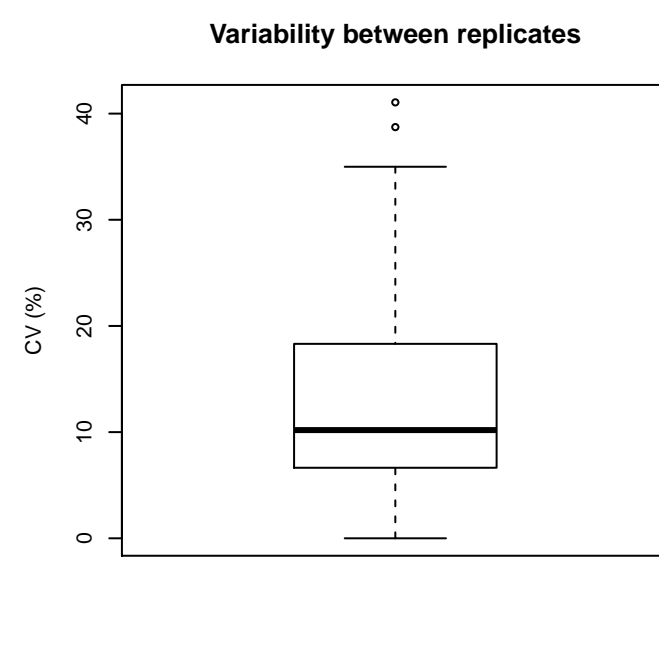

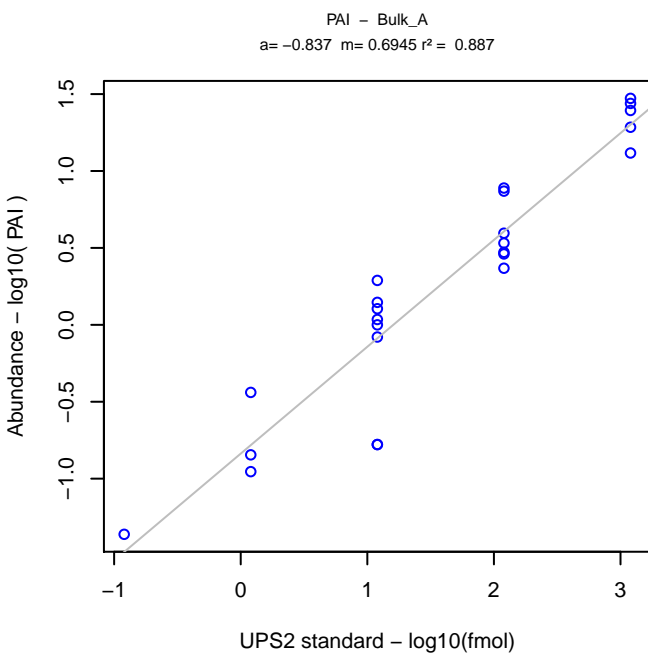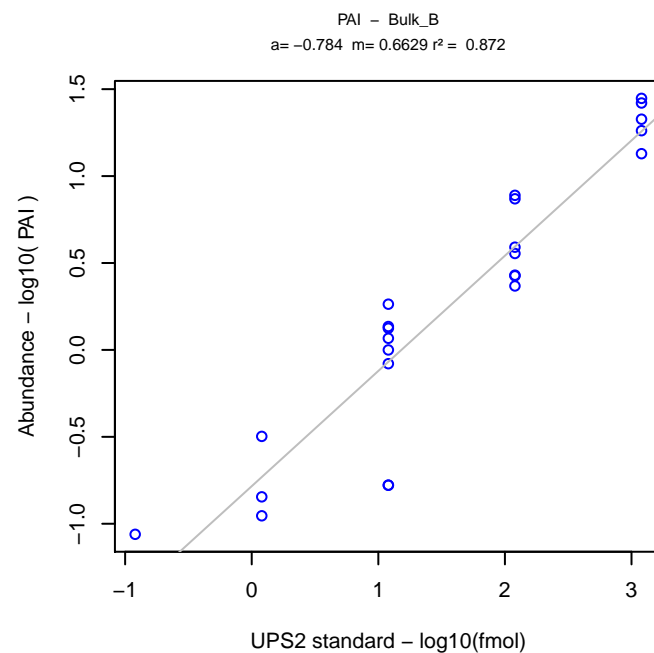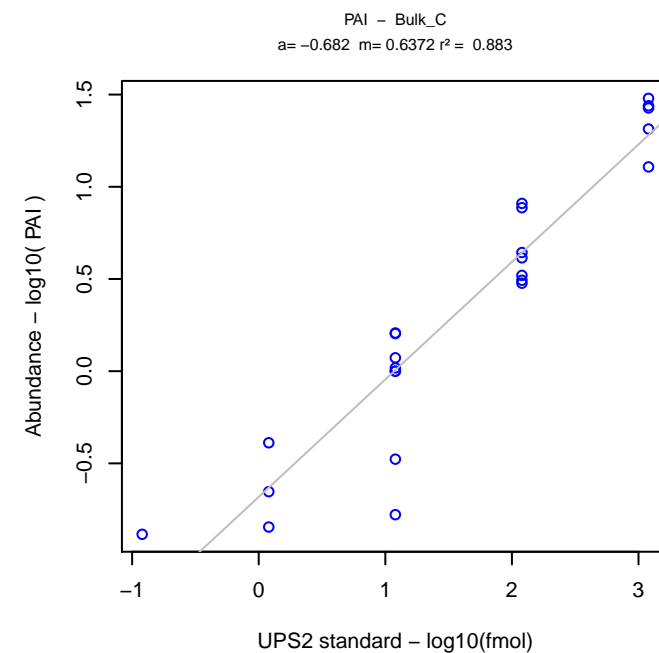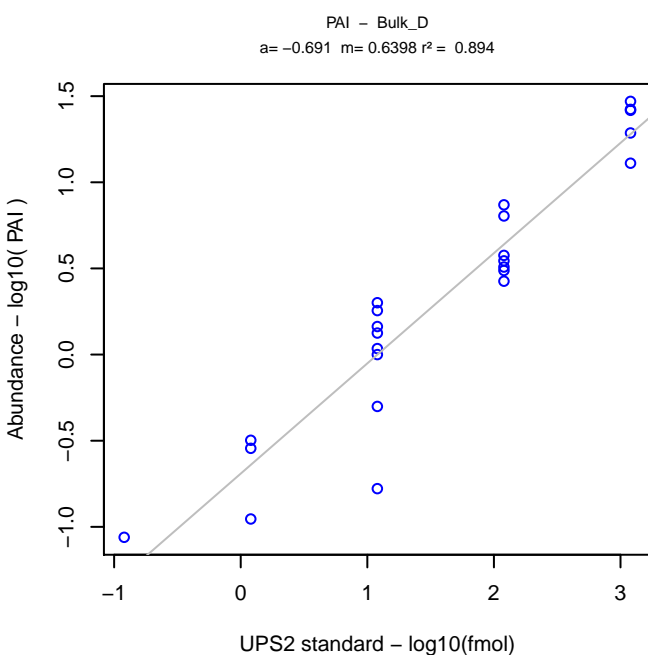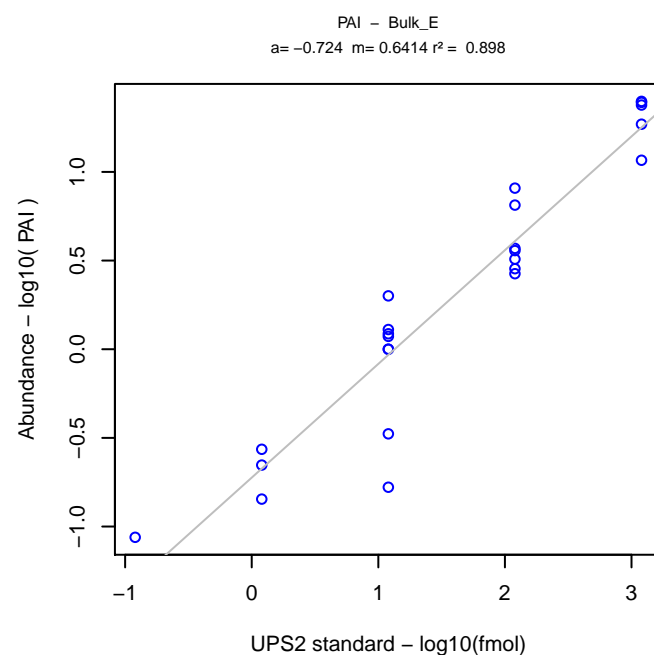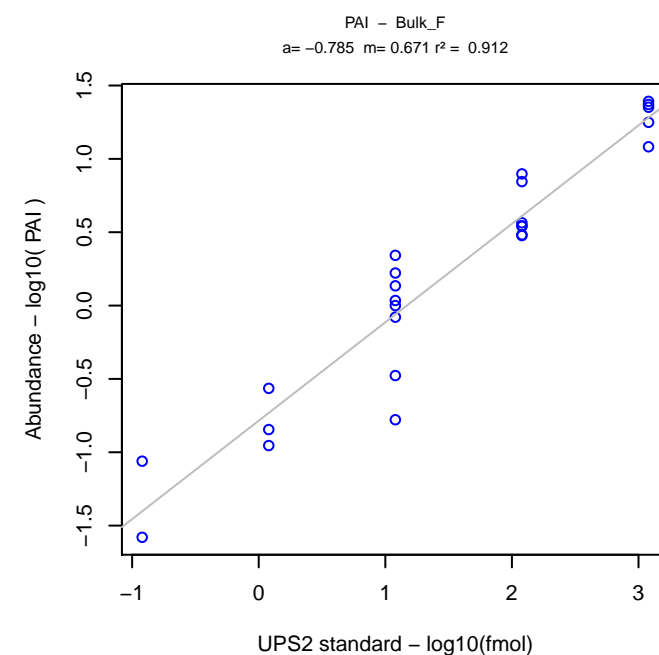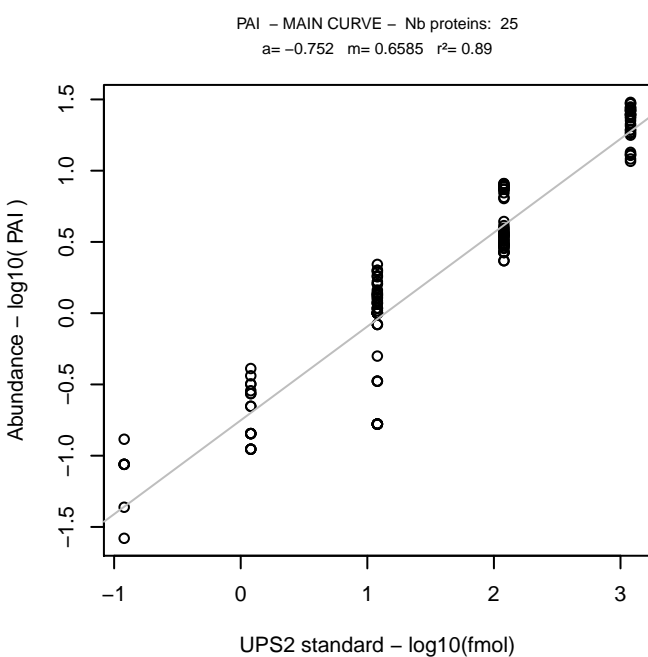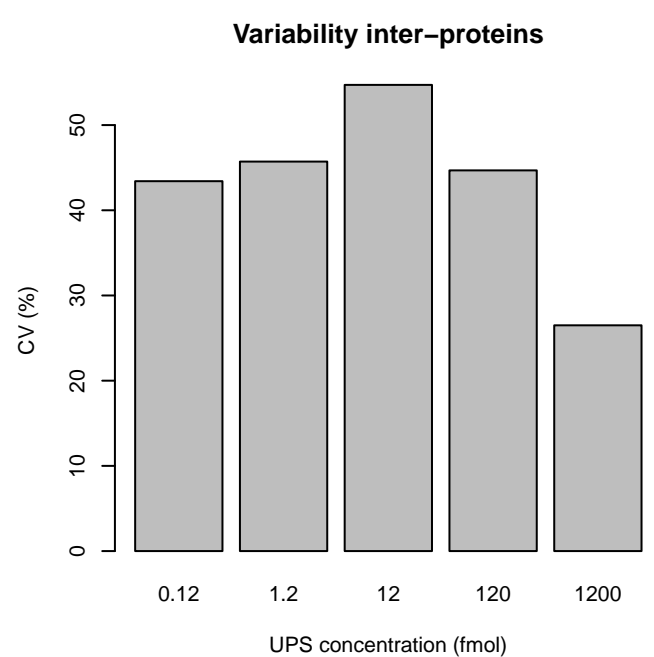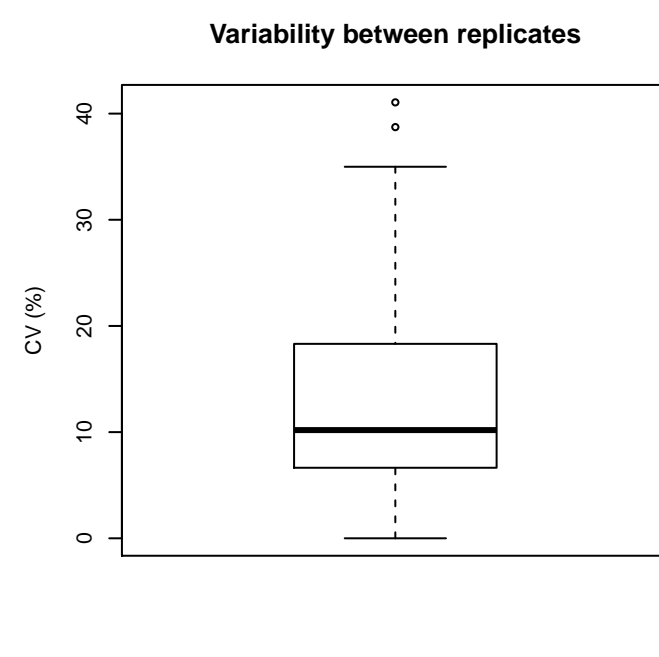

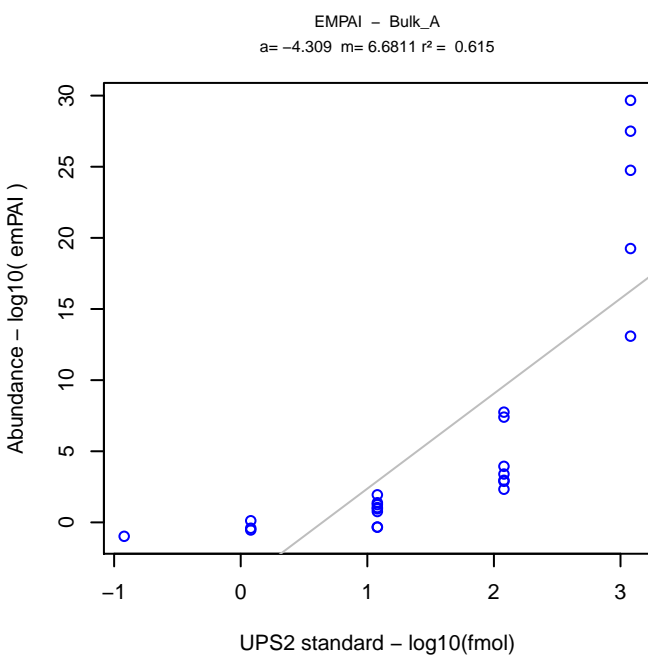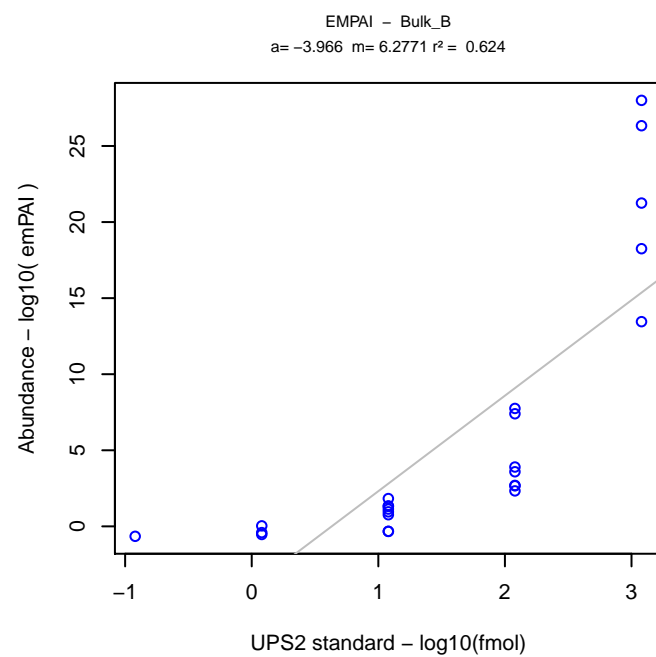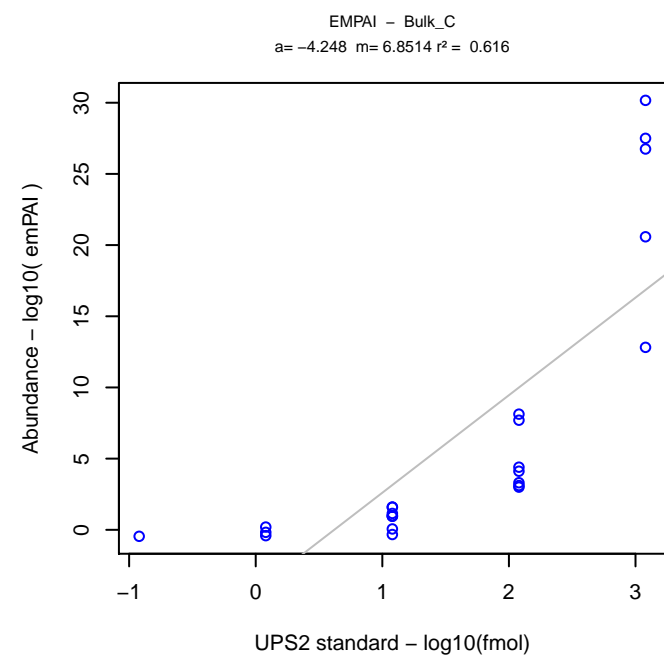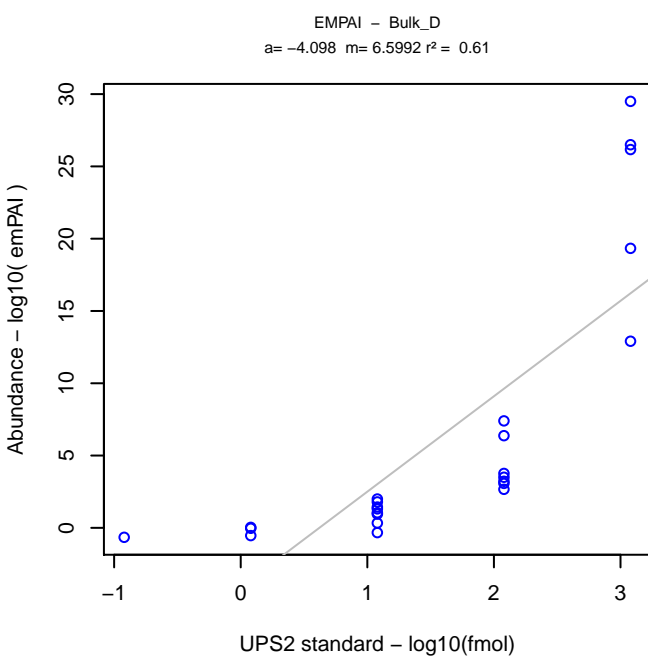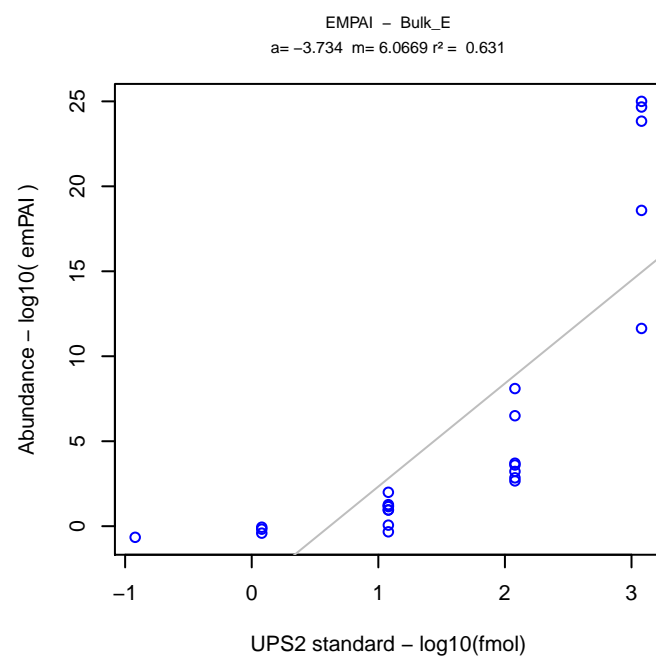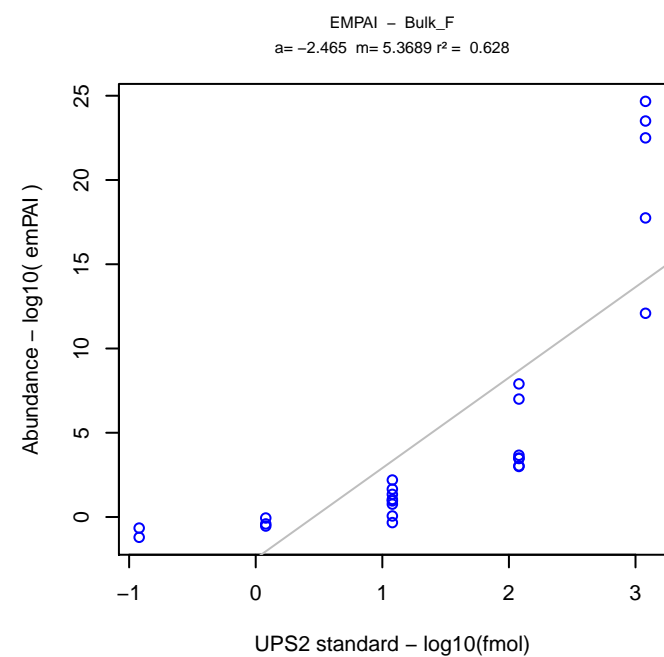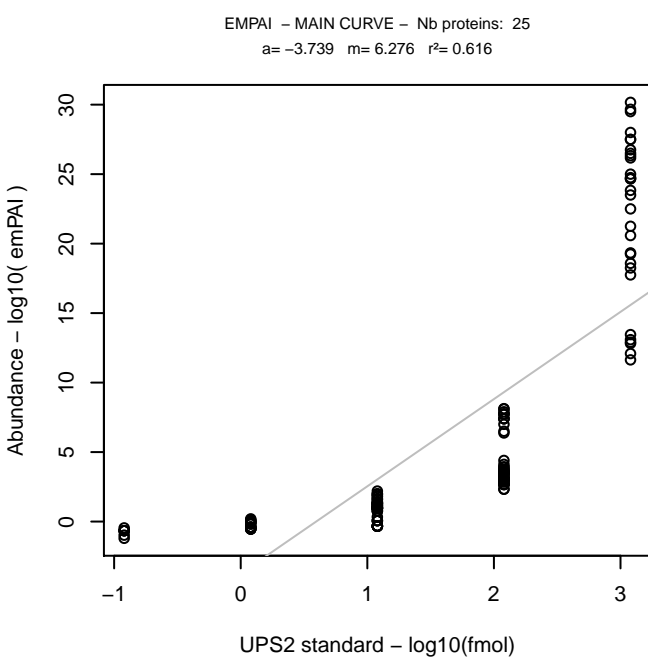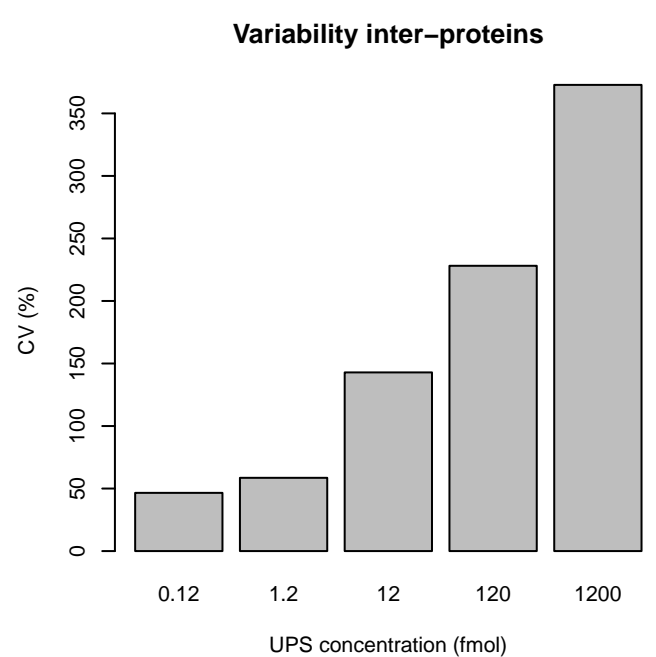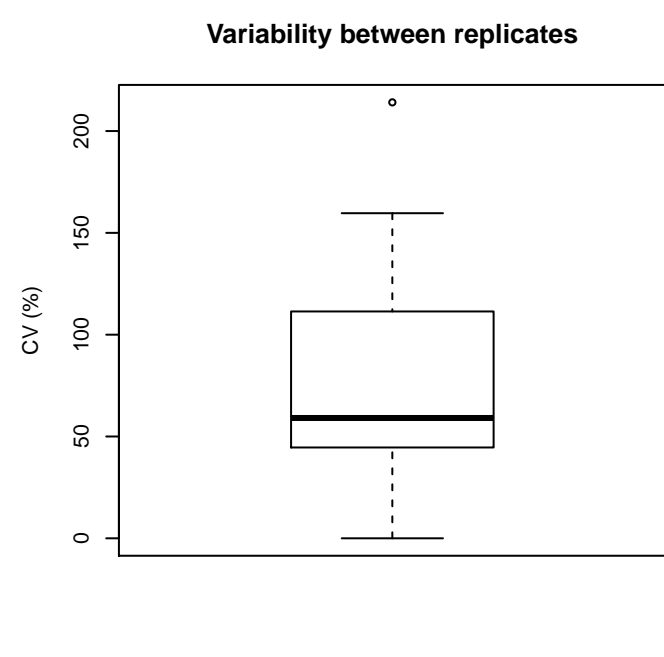

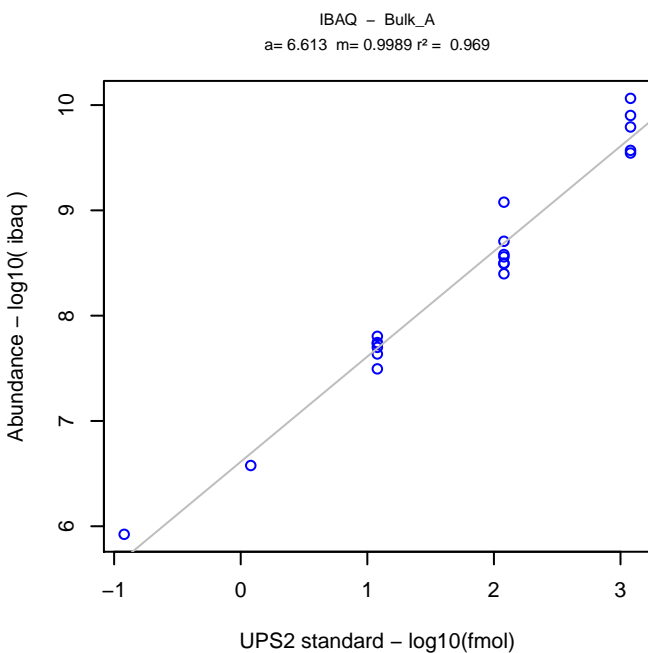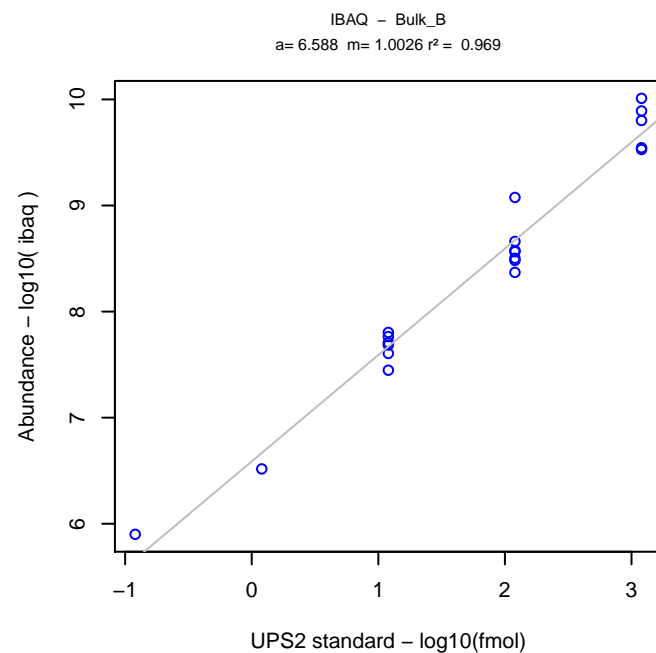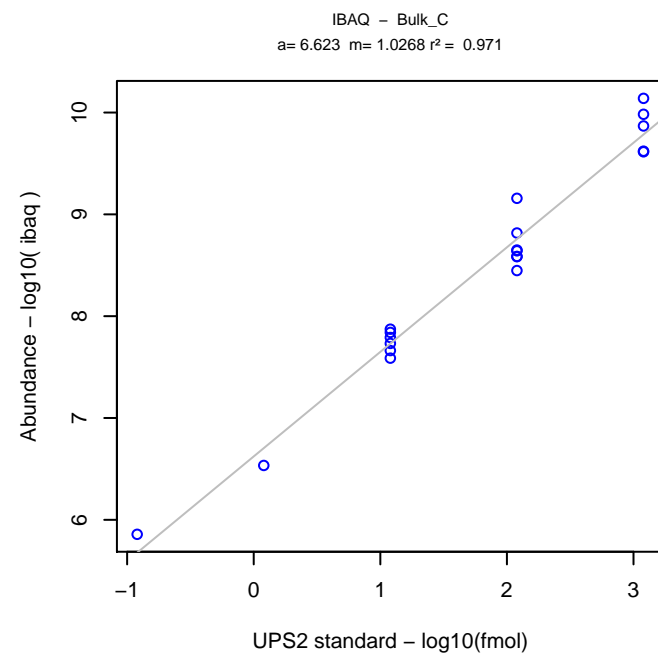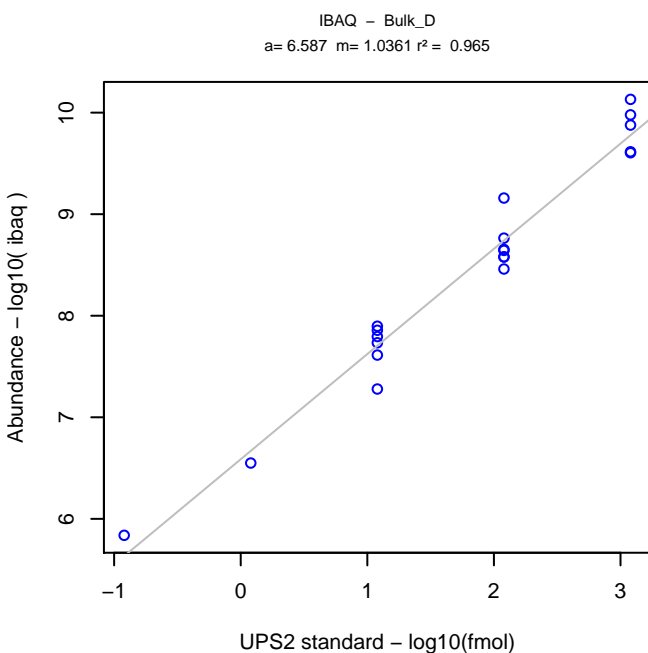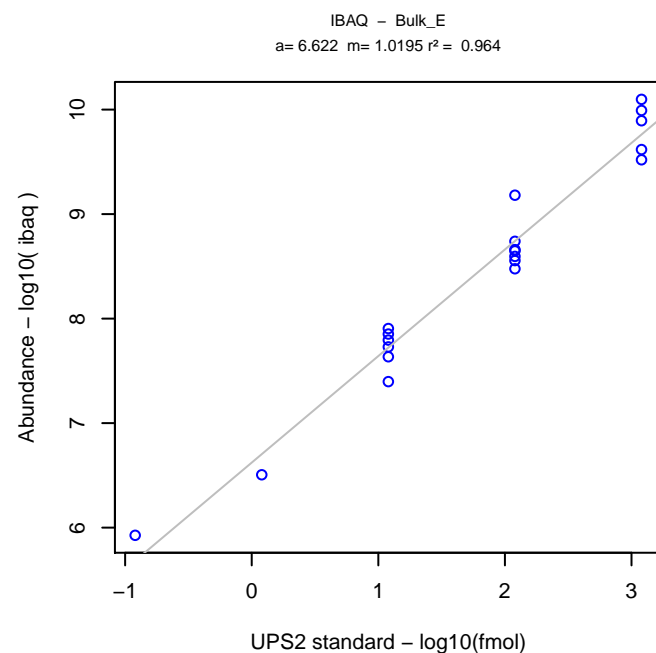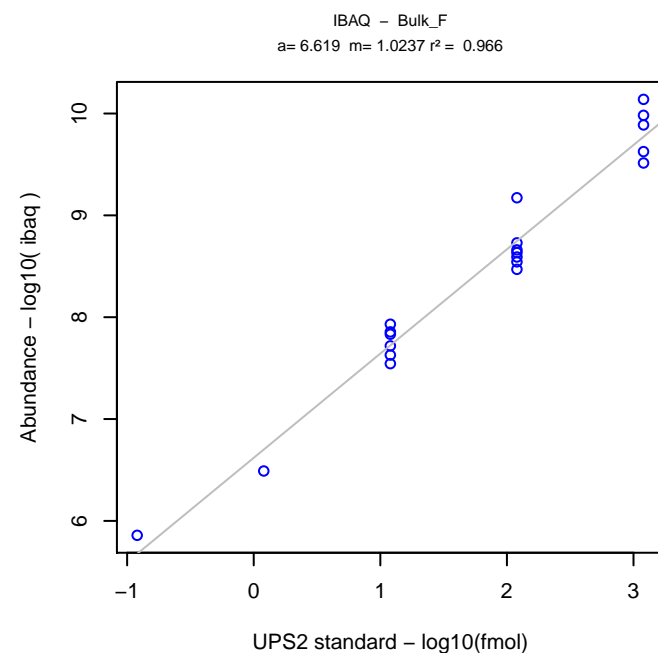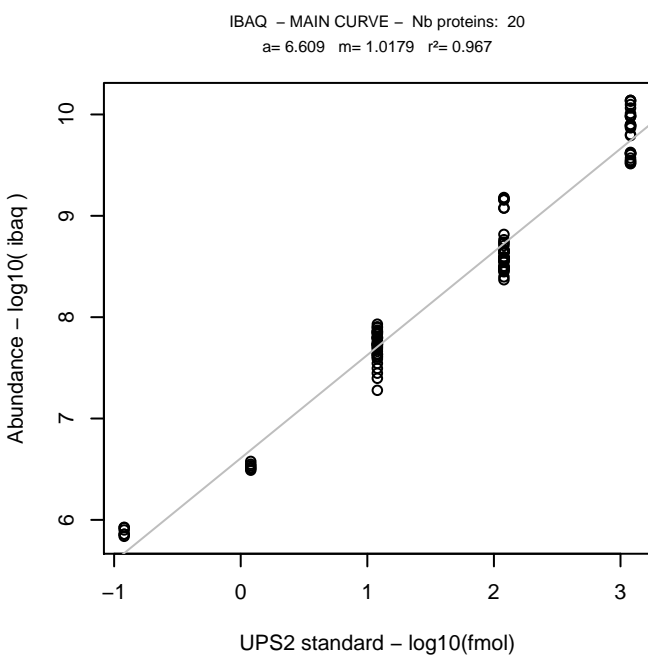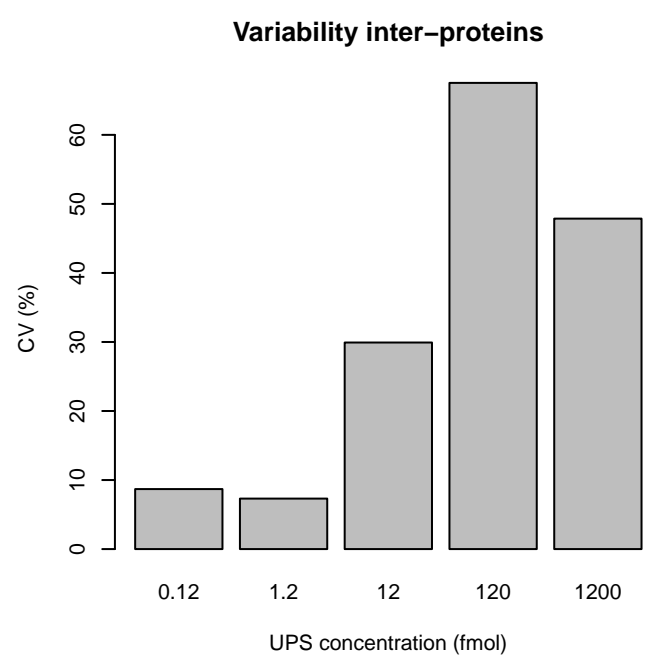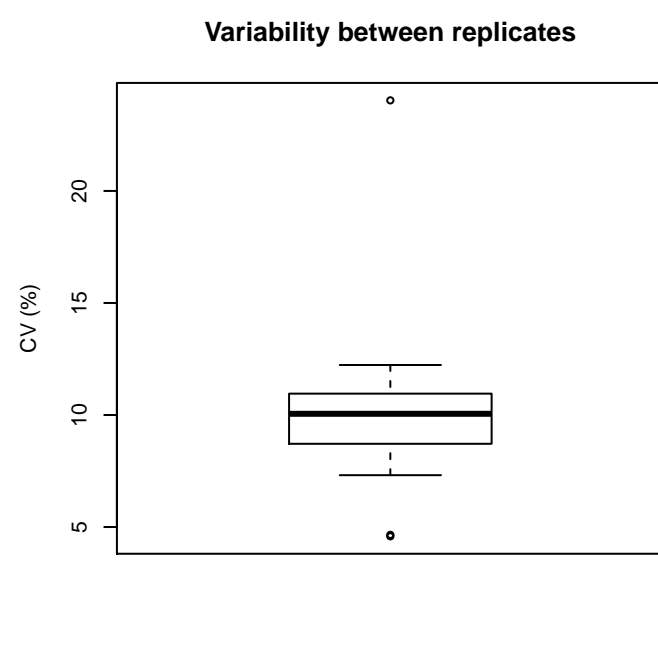

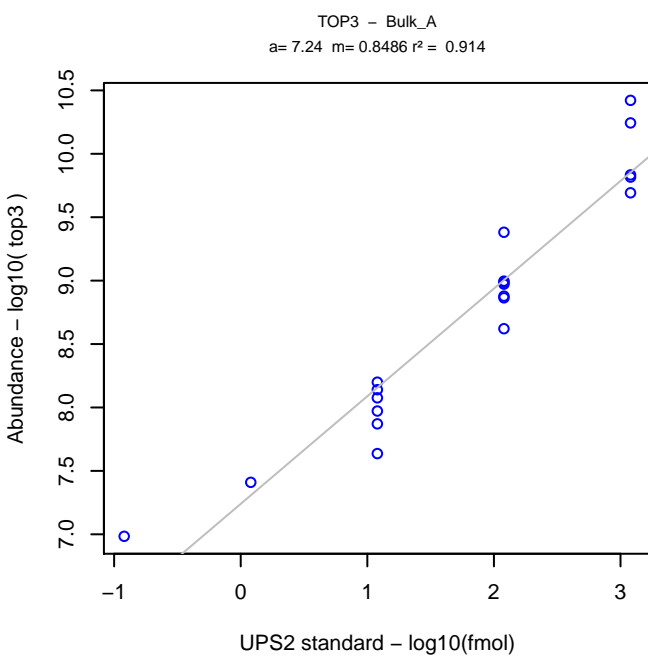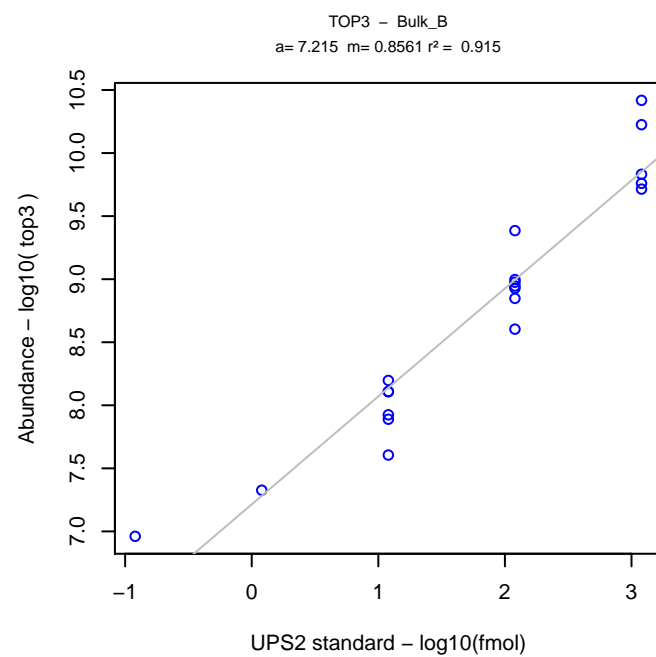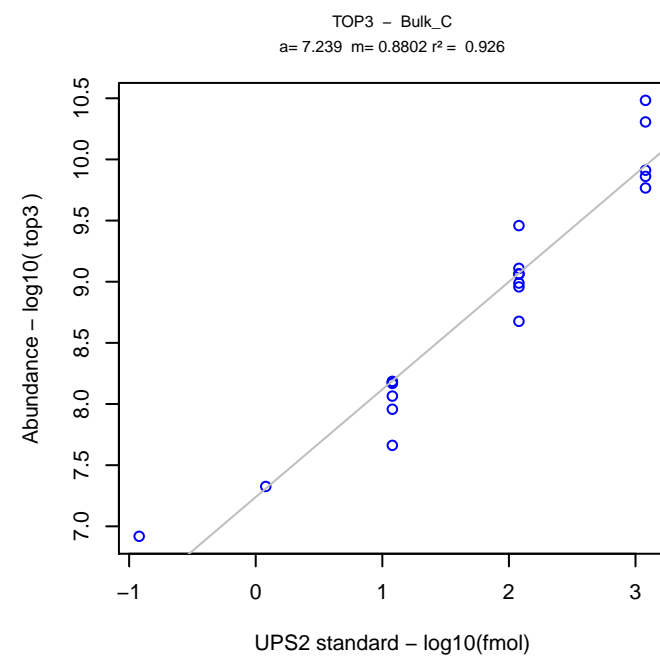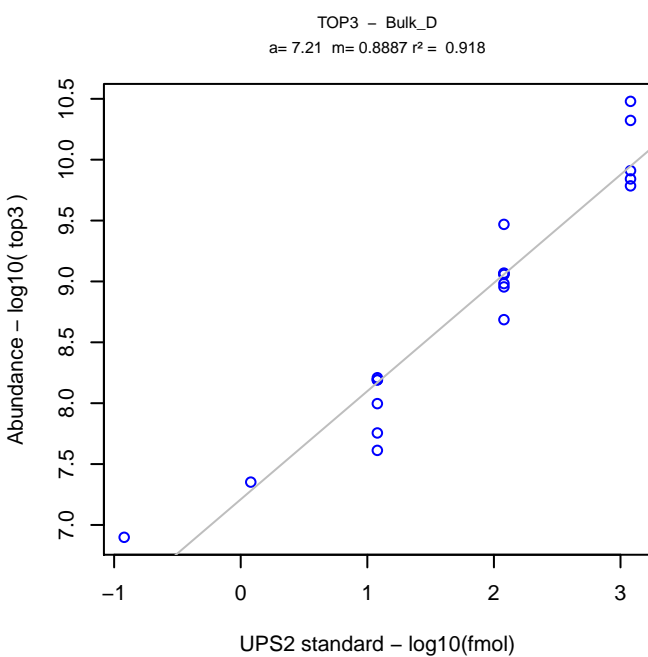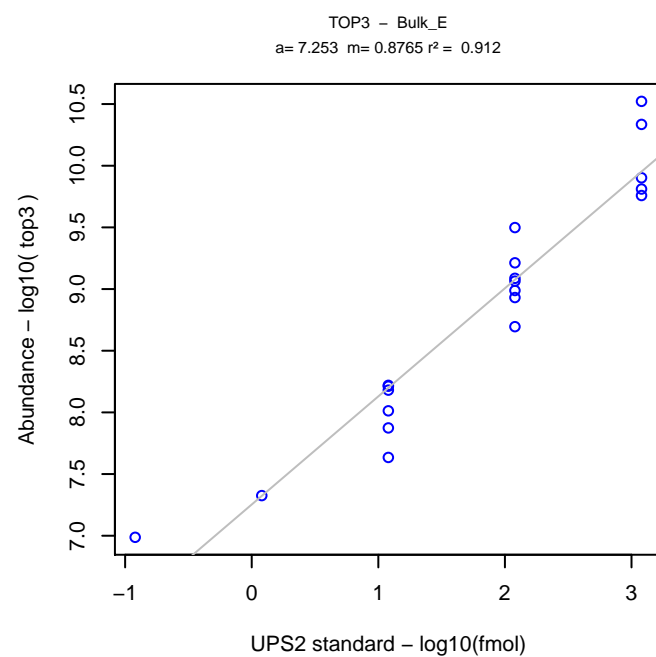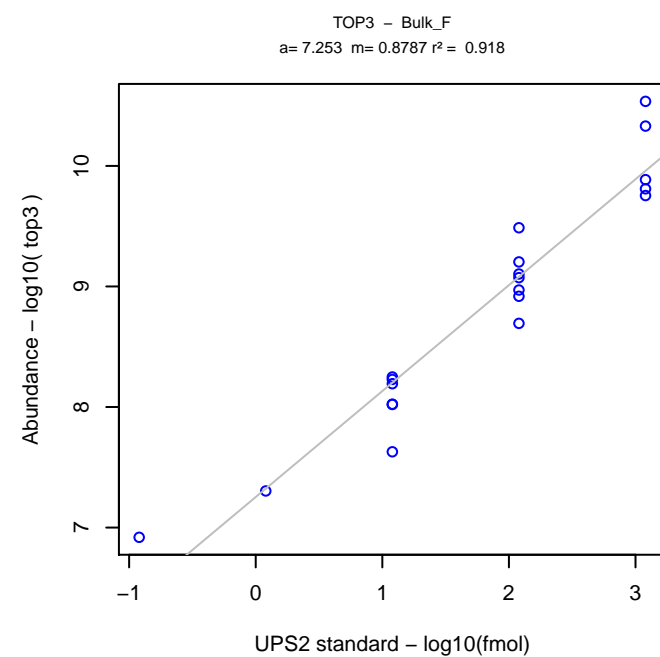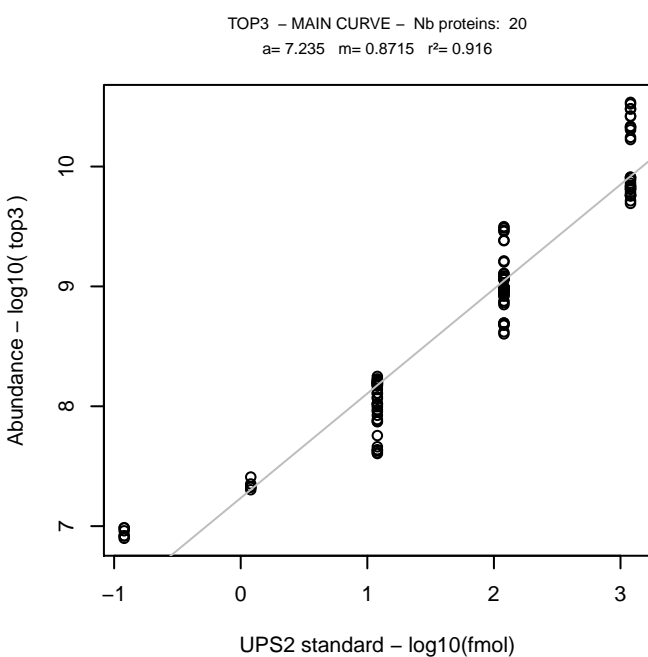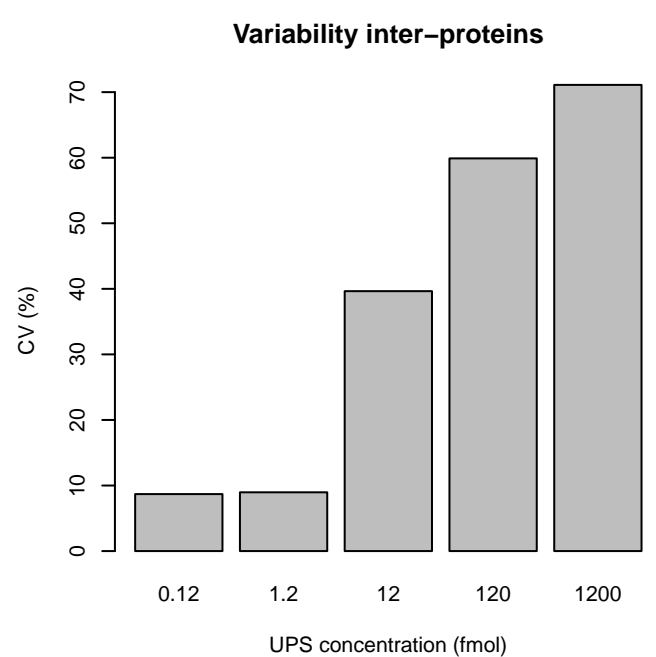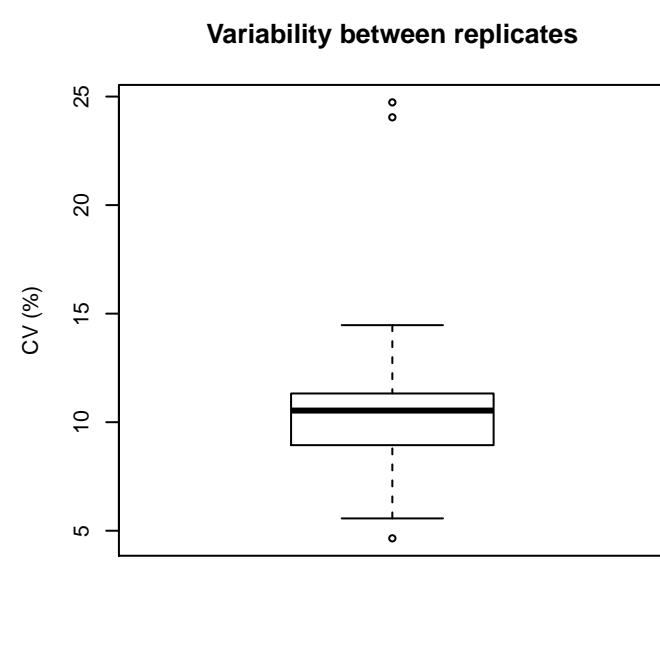

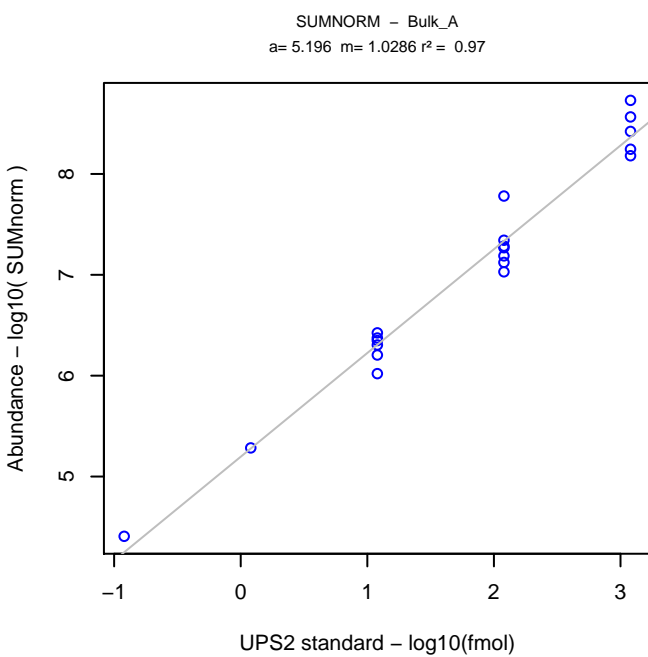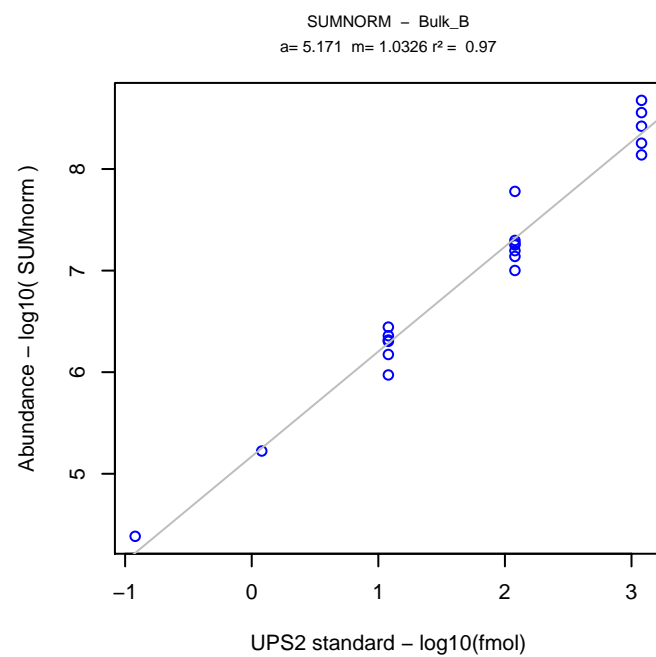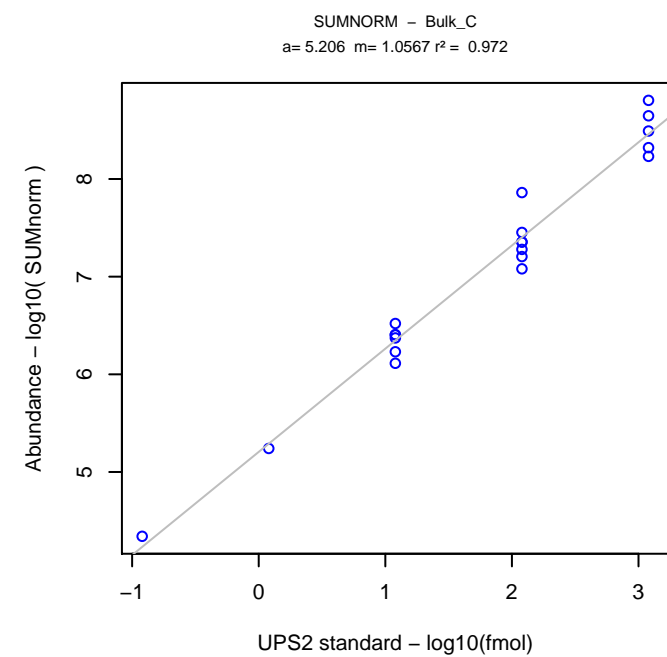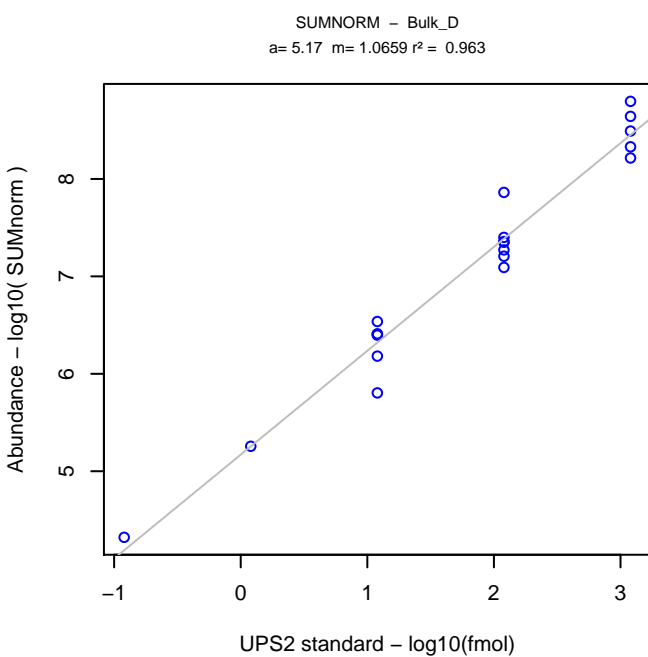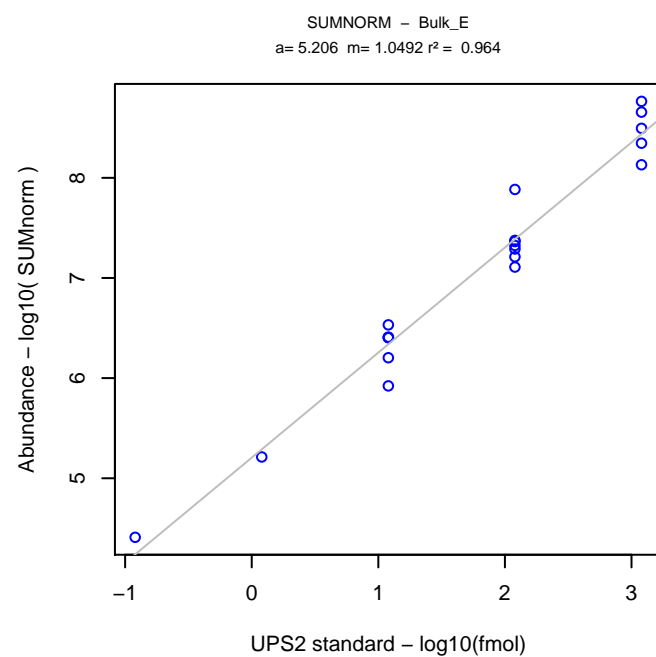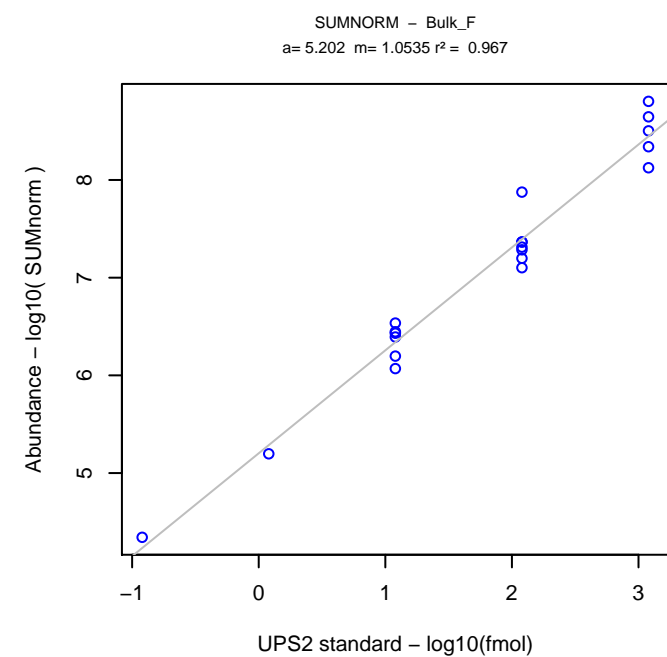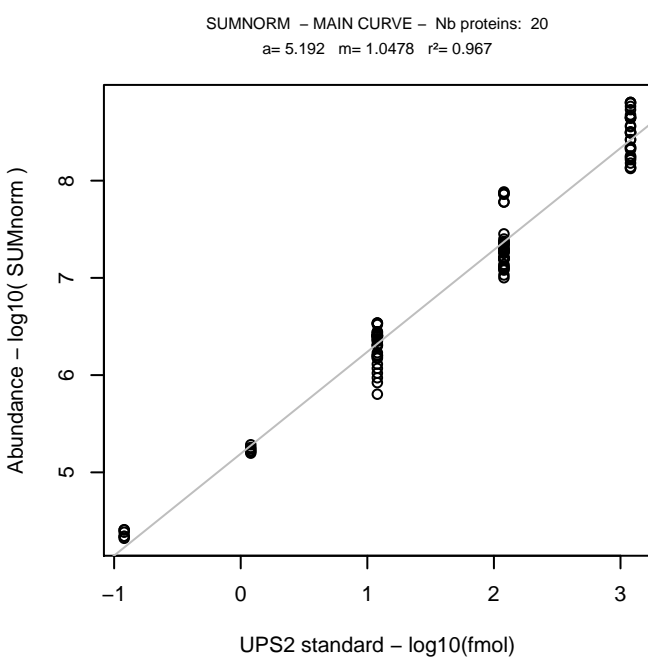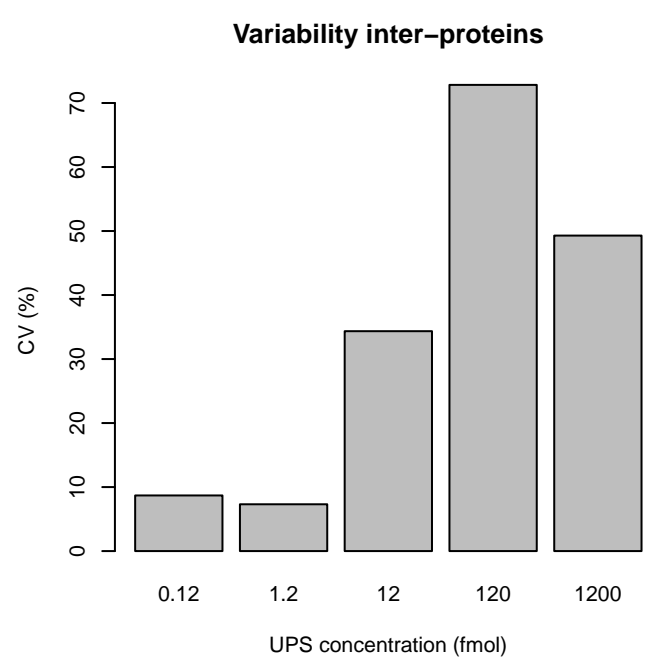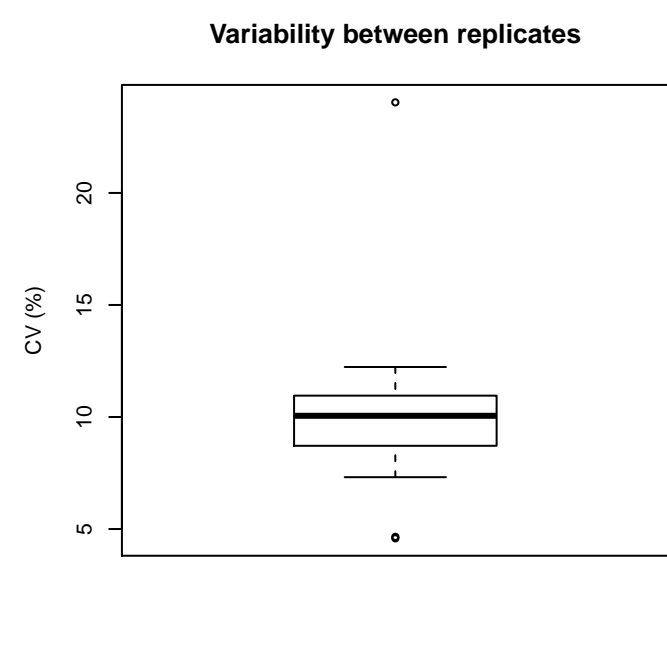

Supplement: Supplementary file 1 [file proteomes-10-00002-s001.zip › FileS2_Linear_regressions.pdf]
